# The Role of Midwives in US Perinatal Palliative Care: A Scoping Review

**DOI:** 10.1111/jmwh.13664

**Published:** 2024-07-09

**Authors:** Robyn Schafer, Jenna A. LoGiudice, Pamela Hargwood, Abigail Wilpers

**Affiliations:** ^1^ Division of Advanced Practice Rutgers University School of Nursing Newark New Jersey; ^2^ Department of Obstetrics, Gynecology, and Reproductive Sciences Rutgers Robert Wood Johnson Medical School New Brunswick New Jersey; ^3^ Fairfield University Egan School of Nursing and Health Studies Fairfield Connecticut; ^4^ Rutgers University Robert Wood Johnson Library of the Health Sciences New Brunswick New Jersey; ^5^ Department of Family & Community Health University of Pennsylvania School of Nursing Philadelphia Pennsylvania; ^6^ Research Institute Children's Hospital of Philadelphia Philadelphia Pennsylvania

**Keywords:** life‐limiting fetal condition, midwifery education, midwifery, perinatal hospice, perinatal loss, termination of pregnancy for fetal anomalies (TOPFA)

## Abstract

**Introduction:**

Perinatal palliative care (PPC) is a rapidly growing and essential reproductive health care option for pregnant persons with a diagnosed life‐limiting fetal condition who continue their pregnancy. The provision of PPC is within the scope of basic midwifery competencies, and midwives are well‐positioned to make unique and valuable contributions to interprofessional PPC teams. However, little is known about midwives’ past or current involvement in PPC in the United States.

**Methods:**

This scoping review of the literature investigated what is known about the role of midwives in PPC in the United States. Multiple databases of published literature were used for this review: PubMed, CINAHL, Embase, Web of Science, ProQuest, Google Scholar, and relevant citations from identified studies. All types of English language publications addressing midwives’ involvement in PPC in the United States were included, without any limitations on publication date.

**Results:**

The role and contributions of midwives in PPC is not well represented in existing literature. Of the 259 results identified, 7 publications met criteria for inclusion. These included 5 case reports, one quantitative research article, and one conference abstract. Midwives are involved in PPC through the provision of direct clinical care (including antepartum, intrapartum, postpartum, neonatal, bereavement, postmortem, and follow‐up care) and care planning and coordination as part of an interprofessional team.

**Discussion:**

Despite midwives being uniquely positioned to provide holistic, family‐centered, and person‐centered care in situations of pregnancy with life‐limiting fetal conditions, there is limited literature about their involvement in PPC in the United States. PPC should be incorporated into midwifery education and training programs. Midwives should play a central role in shaping future research and policies to ensure the accessibility and quality of PPC.

## INTRODUCTION

Perinatal palliative care (PPC) is a crucial aspect of reproductive health care for pregnancies with a diagnosed life‐limiting fetal condition (LLFC). Life‐limiting conditions are those that have no cure and are very likely to result in pregnancy loss, stillbirth, or neonatal death.^1^ These conditions include chromosomal abnormalities such as trisomy 13 or 18 and congenital malformations such as anencephaly, renal agenesis, congenital diaphragmatic hernia, and severe cardiac defects.^2^, ^3^ The profound psychological and emotional impact of an LLFC diagnosis and the expected perinatal loss necessitates specialized care throughout pregnancy and postpartum and neonatal periods.^4^ PPC is one option along the spectrum of reproductive health care for LLFCs that includes termination of pregnancy (abortion care), maternal‐fetal surgery, and life‐prolonging neonatal interventions.^5^

  

Continuing education (CE) is available for this article. To obtain CE online, please visit http://www.jmwhce.org. A CE form that includes the test questions is available in the print edition of this issue.

When a person elects to continue a pregnancy with a known LLFC, a multispecialty care team is necessary to facilitate complex perinatal clinical decision‐making and care coordination. As an interprofessional, holistic approach to care, PPC aims to minimize suffering and maximize quality of life in accordance with the family's values and preferences.^3^, ^5^ Palliative care encompasses more than just hospice or comfort care (see Table 1). It extends throughout the perinatal course and across care settings including ambulatory care clinics and practices, hospitals, and community‐based care (ie, homes).^6^ PPC may be provided concurrently with life‐prolonging interventions and incorporates the full care spectrum such as testing and diagnosis, options counseling, prenatal care, specialty consultations, advanced care planning, intrapartum and postpartum care, neonatal end‐of‐life and postmortem care, and support for the educational, psychological, emotional, and spiritual needs of the bereaved family.^7^ Because health care providers may be deeply affected by complex end‐of‐life decisions and the death of a neonate, PPC can also include support for involved health professionals.^8^

**QUICK POINTS**

✦ Perinatal palliative care (PPC) is a specialized approach to care for persons who continue a pregnancy with fetal conditions that have no cure and are likely to result in pregnancy loss, stillbirth, or infant death.
✦ As part of an interprofessional care team, midwives partner with individuals and families to develop personalized, carefully coordinated, and compassionate care plans to meet their needs throughout the perinatal period and minimize suffering and maximize quality of life for the newborn after birth.
✦ Providing perinatal care and referrals for pregnancies with a fetus with a life‐limiting condition is a core competency of midwifery care in the United States, and midwifery education programs must provide opportunities for students to gain knowledge and skills related the provision of PPC.
✦ Midwives should play a central role in shaping policies affecting individuals and families facing life‐limiting fetal conditions, especially in setting quality standards for PPC and addressing misrepresentations of such care in antiabortion legislation.
✦ There is limited literature about midwives’ involvement in PPC in the United States; future research is needed to guide interprofessional clinical practice, midwifery education, and health policy recommendations.

**Table 1:** Perinatal Palliative Care Terminology

Term	Meaning
Bereavement care	Support provided to individuals and families who have experienced a loss.
Comfort care	Care given to people who are near the end of life and have stopped treatment to cure or control their disease, with a focus on providing comfort, quality of life, and dignity.
Hospice care	Specialized support for end‐of‐life care; a component of PPC.^a^
LLFC	Fetal conditions for which there are no cure and little or no prospect of long‐term ex utero survival without severe morbidity or extremely poor quality of life.
Neonatal death	Death of a newborn in the first 28 days of life.
PPC	Specialized care throughout the perinatal period (including pregnancy, intrapartum, postpartum, newborn, and postmortem) for individuals who continue a pregnancy with a known LLFC.
Pregnancy loss	Broadly refers to any situation where a pregnancy does not result in the birth of a live newborn.
Stillbirth	When a fetus dies after 20 weeks of pregnancy, prior to or during birth, showing no signs of life and is unable to be resuscitated.

Abbreviations: LLFC, life‐limiting fetal condition, PPC, perinatal palliative care.

a Although this term is sometimes used interchangeably with PPC, PPC offers a wider range of services than hospice care and can be provided along with curative or life‐prolonging treatments in addition to hospice care.

Adapted from the Centers for Disease Control and Prevention, the American College of Obstetricians and Gynecologists, the March of Dimes, and the Eunice Kennedy Shriver National Institute of Child Health and Human Development.

In the last decade, this emerging area of care has witnessed significant growth as advances in prenatal diagnostic tools have enhanced detection of LLFCs and a strong body of evidence that has shown that standard perinatal care fails to address the needs of patients and families in these unique cases.^9^, ^10^, ^11^, ^12^, ^13^, ^14^ Globally, referrals for PPC are increasing, and the number of PPC programs in the United States is also growing, from only 75 identified programs in a 2016 study to more than 250 hospitals, hospices, and community‐based organizations reported on an online registry (perinatalhospice.org) in 2024.^15^, ^16^ The evolution of PPC signifies widespread acknowledgment of its importance to families and underscores the need for increased training for perinatal health care professionals to provide such care.

High‐quality PPC requires a comprehensive, team‐based, coordinated approach,^5^, ^17^, ^18^, ^19^ and midwives are uniquely positioned to be valuable members of the PPC team. Many midwives in the United States are trained as nurses and work in collaboration with obstetrician‐gynecologists, neonatologists, and other perinatal care providers. However, midwifery stands as a distinct profession from nursing, obstetrics, or medicine, with its own valuable contributions to PPC.^20^

Even where scope of practice overlaps with other disciplines, midwifery has a unique approach to care and underlying philosophy.^21^ The International Confederation of Midwives recognizes that midwifery “has a unique body of knowledge, skills and professional attitudes” grounded in “a professional framework of autonomy, partnership, ethics and accountability.”^22^ Person‐centered care, promotion of continuity of care, and the facilitation of healthy family and interpersonal relationships are among the Hallmarks of Midwifery, in alignment with the individualized, relationship‐oriented, family‐centered approach that PPC requires.^15^, ^23^, ^24^, ^25^ Similarly, the midwifery model of care embraces qualities central to PPC such as a continual and compassionate patient‐provider partnership, collaborative interprofessional care, nonintervention in normal physiologic processes, therapeutic value of human presence, and skillful communication.^5^, ^26^, ^27^, ^28^, ^29^ With experience in caring for people during important life transitions including perinatal loss, midwives are well equipped to provide psychosocial support and compassionate palliative and bereavement care.^30^ In addition, certified nurse‐midwives and certified midwives in the United States have an expansive scope of practice that extends throughout pregnancy, intrapartum, and postpartum into primary care of the healthy newborn and across hospital, ambulatory, and community settings.^31^ As such, midwives have the potential to enhance PPC teams in ways distinct from nursing, obstetric, or pediatric care providers.

The provision of PPC is within the Core Competencies of Basic Midwifery Practice in the United States, as defined by the American College of Nurse‐Midwives (ACNM).^23^ In 2020, ACNM extended the scope of midwifery care from “appropriate interventions and referrals for abnormal conditions… [including] end‐of‐life care for stillbirth and conditions incompatible with life,” to “palliative care for conditions incompatible with life, including addressing the psychosocial needs of a grieving parent.”^23^, ^32^ Midwifery educators are working to adequately integrate PPC into their curricula.^33^, ^34^ The need for PPC providers has expanded following recently instituted restrictions on abortion care that include pregnancies with LLFCs in several states. There is evidence that some midwives are less comfortable than physicians in providing or facilitating PPC.^35^ Research is needed to assess US midwives’ involvement in PPC to optimize health care quality and outcomes. The aim of this scoping review was to identify what is known about the role of midwives in PPC in the United States and highlight implications for clinical practice, midwifery education, health policy, and research.

## METHODS

This study used a scoping review design to create a comprehensive overview of research on midwives involved in PPC in the United States. A scoping review method was determined to be most appropriate since the role of midwives in PPC is an area of emerging research which can benefit from identification of available evidence and gaps in the existing literature.^36^ The Population, Concept, Context framework^37^ was used to guide our search to investigate the research question: what is known about midwives’ (population) role in PPC (concept) in the United States (context)?

### Search Strategy

Preliminary searches were undertaken in multiple databases (JBI Evidence Synthesis, Cochrane Database of Systematic Reviews, CINAHL, PubMed, and Epistemonikos) to determine if there were any existing systematic or scoping reviews on this topic; none were identified. The search strategy for this review (Supporting Information: Appendix S1) was developed in consultation with a research librarian with expertise in systematic review methodology. It included terms specific to midwives and perinatal palliative or hospice care and aimed to locate both published and unpublished studies through a threefold approach of searching (1) academic databases, (2) gray literature sources, and (3) references in identified publications. To ensure comprehensive results, terms specific to location (ie, United States) were not included; instead, articles were screened for context during the review process.

Searches were conducted in the following electronic databases: PubMed, CINAHL (EBSCO), Embase, Web of Science, and ProQuest (Dissertations and Theses Global) through December 19, 2023. In addition, the first 100 results from a Google Scholar search were included, as were results of citation searching from publications identified through this search strategy. Results were limited to English language. No time limitations were applied.

The initial search yielded 230 records published from 1992 to 2024, including quantitative, qualitative, and mixed methods research; reviews of the literature; study protocols; case reports; descriptions of clinical models, programs, or guidelines; synopses of recent publications; editorials and commentaries; poster and conference abstracts; book chapters in education textbooks; and article errata. Hand‐searching of citations from literature reviews, book chapters, and other relevant publications resulted in an additional 29 records. After 17 duplicate results were removed, 242 records were entered into Endnote (reference management software) and imported into Covidence (systematic review software) for review. Titles and abstracts were screened for eligibility by 2 independent and interprofessional reviewers (first and last author).

Except for literature reviews, all types of scholarly publications including commentaries and editorials, case studies, program descriptions, dissertations, and original research were included. Because the focus of this scoping review was midwives’ role in PPC, publications were excluded if they addressed only bereavement or neonatal palliative care or unexpected perinatal loss or contained only vague references to midwives’ involvement. Where it was not possible to determine eligibility criteria (such as an unspecified location), authors were contacted for additional clarifying information.

**Figure 1:**
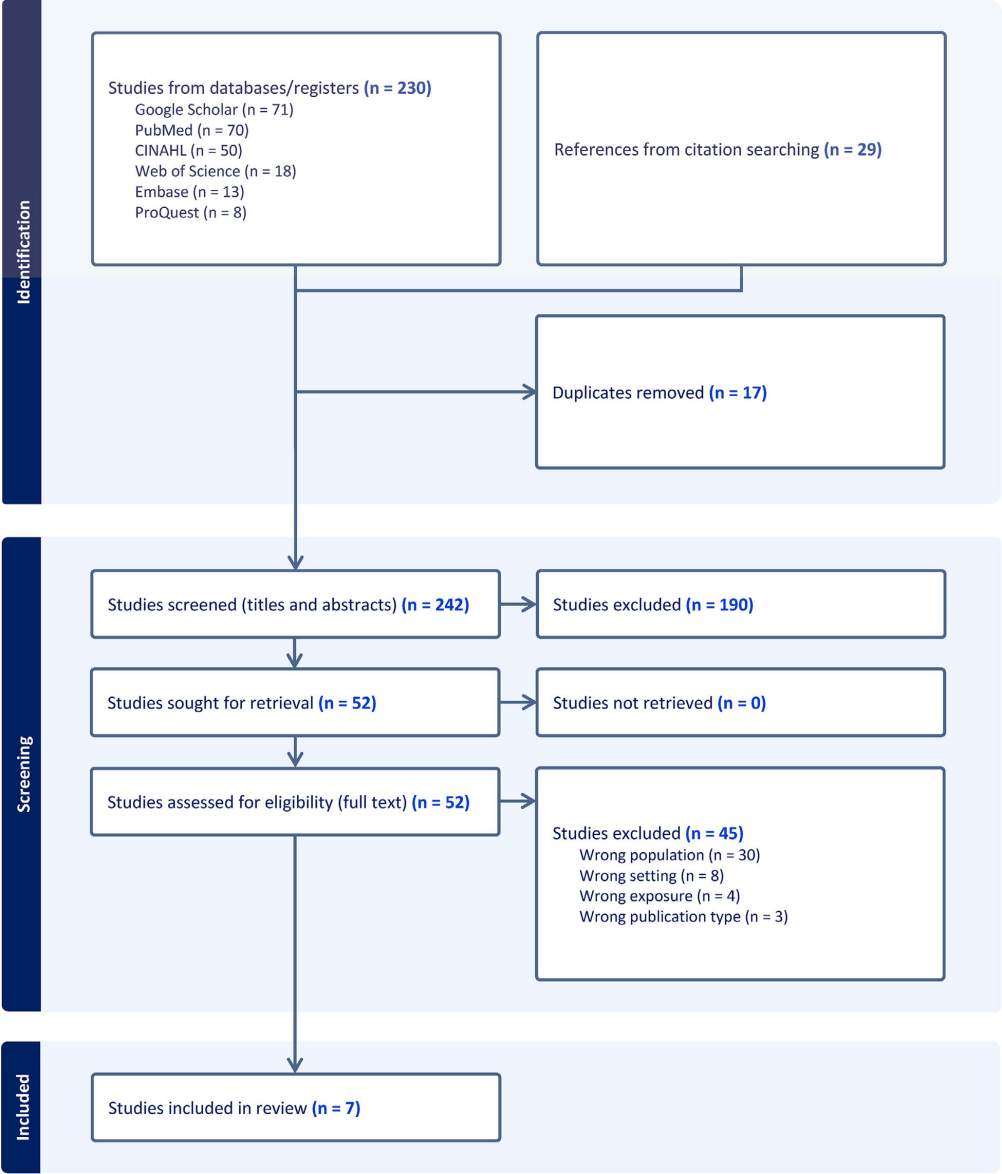
PRISMA Diagram Abbreviation: PRISMA, preferred reporting items for systematic reviews and meta‐analyses.

In total, 52 records were examined in full text review by multiple researchers (first, second, and last author). Records were excluded during screening for the following reasons: wrong population (not midwives) (n = 30), wrong location (outside the United States) (n = 8), wrong exposure (not PPC) (n = 4), and wrong publication type (eg, book chapter, literature review) (n = 3). Any discrepancies in screening were discussed and resolved by consensus, resulting in a total of 7 studies that met inclusion criteria. Data extraction was conducted by the first author, confirmed by second and last authors, and summarized into tables for data synthesis and presentation (see Table 2 and Table 3). Guidelines for preferred reporting items for systematic reviews and meta‐analyses extension for scoping reviews (PRISMA‐ScR) were followed (Supporting Information: Appendix S2).^38^ A PRISMA flow diagram is shown in Figure 1. Included articles were not appraised for methodological quality or risk of bias, as such efforts are not aligned with the study aim or scoping review methodology.^37^

**Table 2:** Summary of Included Publications

Publication Authors (Year)	Objective	Design	Location	Summary of Midwife's Role in PPC
Cole et al^40^ (2018)	Examine milk donation in a PPC program.	Case report; program description of clinical counseling and care for milk donation with an anticipated perinatal loss for LLFC.	High‐risk fetal center within a specialty pediatric hospital (Fetal Diagnosis and Treatment at the Children's Hospital of Philadelphia, Pennsylvania).	Midwives and other advanced practice providers such as women's health nurse practitioners play a core role in PPC by obtaining a detailed health history, orienting patients to the center, providing antepartum care and education, and coordinating care including a consultation for lactation and postpartum milk donation.
English and Hessler^24^ (2013)	Describe advance‐care birth planning at a PPC center.	Case report; description of a family's experience in a PPC service.	High‐risk, maternal‐fetal newborn center (Fort Collins, Colorado).	As primary prenatal care providers, midwives contribute to the creation of a PPC advance‐care birth plan through shared decision‐making and person and family‐centered interprofessional care.
Kauffman, Hauck, and Mandel^41^ (2010)	Describe a PPC service.	Care report; description of the creation of a PPC program.	Regional, Level III hospital (Lancaster General Women and Babies Hospital, Lancaster, Pennsylvania).	Midwives are external to the PPC team; they attend education sessions, provide referrals to the service, and contribute to development of an individualized pregnancy and birth plan.
Knowles, Vente, and Fry^43^ (2021)	Describe the role of PPC consultation for a planned home birth with a LLFC.	Conference abstract (case report and presentation).	Antepartum consultation in planned home birth (Lurie Children's Hospital of Chicago, Chicago, Illinois).	Midwives are external to the PPC team; families receiving midwifery care for a planned community birth with known LLFCs may consult with PPC to receive home hospice care and develop a contingency plan.
LoGiudice and O'Shea^34^ (2018)	Present a model of PPC education in a midwifery program.	Case report; description of a model of PPC in midwifery education.	Nurse‐midwifery education program (Fairfield University, Fairfield, Connecticut).	Midwifery students who receive PPC education develop knowledge and skills necessary to provide holistic midwifery care to those with a fetus with a LLFC in antepartum, intrapartum, and postpartum periods and clinical care settings.
Wool^42^ (2015)	Measure barriers that physicians and APNs encounter in providing PPC or referrals.	Quantitative research, cross‐sectional survey.	National sample of physicians (n = 66) and APNs (n = 146, including 70 CNMs).	Nurse‐midwives and other APNs report more barriers in providing PPC and referrals and lower levels of confidence in their ability to provide PPC counseling than their physician counterparts.
Wool and Parravicini^39^ (2020)	Describe a PPC program to guide professionals in building a PPC service.	Case report; description of the development of a PPC program.	Large, academic, major metropolitan medical center (Columbia University Irving Medical Center, New York, New York).	Midwives can be part of the interprofessional PPC team by providing perinatal care and education and continuity and coordination of PPC care.

Abbreviations: APN, advanced practice nurse; CNM, certified nurse‐midwife; LLFC, life‐limiting fetal condition; PPC, perinatal palliative care.

**Table 3:** Key Contributions of Midwives in Perinatal Palliative Care

Aspect of Care	Contributions of Midwives
Direct clinical care	Person‐ and family‐centered antepartum, intrapartum, and/or postpartum clinical care, including education and anticipatory guidance.^24^, ^34^, ^39^, ^40^, ^41^, ^42^ Care in community settings (ie, home birth, community‐based hospice).^24^, ^43^ Options counseling and education.^34^ Infant feeding and lactation support, including supporting informed choice for milk donation.^39^, ^40^ Bereavement care, memory‐making, and psychosocial support.^24^, ^34^ Follow‐up after the conclusion of perinatal care (eg, telephone calls, anniversary cards).^34^
Care planning and coordination	Referring pregnant persons with LLFCs to PPC.^42^ Conducting intakes and orienting patients to the structure and functions of the PPC center.^40^ Providing specialized childbirth education.^39^ Collaborating in the development of an advance‐care birth plan and supporting birth plan decision‐making.^24^, ^34^, ^41^ Facilitating communication across the perinatal care team and serving as a liaison to parents and health care providers.^39^, ^40^ Coordinating care and ensuring continuity of care throughout the perinatal course, including consultations with or referrals to specialists such as maternal‐fetal medicine, perinatology, genetics, and ultrasonography.^24^, ^40^ Providing education resources and referrals to support groups and organizations.^34^ Facilitating cultural, religious, and spiritual practices for perinatal death (eg, baptism, naming ceremony).^34^

Abbreviations: LLFC, life‐limiting fetal condition; PPC, perinatal palliative care.

## RESULTS

This scoping review identified 7 publications that addressed midwives’ role in PPC in the United States (see Table 2). These publications included 5 case reports, one quantitative research study, and one conference abstract. Of the 5 case reports, 3 described PPC programs (one in New York^39^ and 2 in Pennsylvania^40^, ^41^), and one presented a model of PPC in a midwifery education program^34^ (Connecticut). The remaining case report^24^ described the experience of a family who received PPC (Colorado) to explore the role of advanced care birth planning in pregnancies with LLFCs. The quantitative study^42^ was a national survey of physicians and advanced practice nurses (APNs) (n = 212, including 70 certified nurse‐midwives) that assessed barriers in providing or referring for PPC. The final publication included in this review was a conference abstract^43^ that presented a case of an antenatal PPC consultation (Illinois) for a planned home birth with a LLFC. Authors’ disciplinary orientations were primarily nursing^24^, ^34^, ^40^, ^41^, ^42^, ^43^ and also included midwifery,^34^, ^40^ neonatology,^43^ and psychiatry.^40^

Included publications describe midwives being involved in PPC programs to widely varying extents, ranging from serving as core members of the PPC team,^39^, ^40^ to filling specific and limited roles,^24^, ^43^ to providing referrals and delegating care to PPC specialists.^41^ In all models, midwives were presented as being a part of (or interacting with) interprofessional PPC teams that included a wide variety of health professionals from different specialties such as nursing, obstetrics and maternal‐fetal medicine, pediatrics and neonatology, genetics, hospice care, child life specialists, lactation, pastoral care, and social work.

Overall, midwives were not well represented in existing literature on PPC. Few studies specifically addressed the role of midwives as part of the PPC team, and identified publications failed to adequately portray midwifery scope of practice or address the unique contributions of midwives as PPC providers. Where midwives were included in the literature, their role in PPC included the provision of midwifery care throughout the perinatal course (including antepartum, intrapartum, postpartum, neonatal, bereavement, postmortem, and follow‐up care) (see Table 3). Midwives were also involved in care planning and coordination such as providing referrals to PPC, conducting intake visits, collaborating in the development of an advanced care birth plan, facilitating communication across the PPC team, coordinating consultations with specialists, and providing education and psychosocial support resources. Midwives reported experiencing barriers in providing PPC and referrals such as a lack of societal understanding and organizational support for PPC, limited time to provide counseling, and low confidence and comfort in providing PPC.^42^ Incorporating PPC into midwifery education was shown to enhance students’ knowledge and skills in providing care to those with a fetus with a LLFC.^34^

## DISCUSSION

This scoping review to investigate what is known about midwives’ role in PPC in the United States identified few publications inclusive of midwives involved in PPC and only one research study. As a relatively new and expanding area of care, PPC research in general is limited and primarily consists of descriptive studies and case reports.^8^ The small number of eligible studies identified in this review is a clear indication of a gap in existing knowledge about midwives’ involvement in PPC in the United States.

Existing literature describes the provision of holistic midwifery palliative care throughout pregnancy, labor/birth, postpartum, and newborn periods in a variety of capacities and roles, including antepartum clinical care and education for a person with a fetus with a life‐limiting condition, coordination of prenatal care and consultations, development of an individualized advanced care birth plan, intrapartum and postpartum care, and bereavement care and memory‐making activities. As part of (or in conjunction with) an interprofessional PPC team, midwives are involved throughout the perinatal course in both the provision of direct clinical care as well as care planning and coordination efforts.

Despite a focus on interprofessional and multispecialty care, PPC teams and programs identified in the literature often failed to include midwives. When they were involved, midwives were often presented as a subset of nursing or interchangeable with other types of advanced practice providers. For example, one publication (excluded for not specifically referencing midwives) stated that “Normalization of the pregnancy is a key component of the care provided by the APRNs during prenatal visits.”^44^ Because the recognition of the normalcy of pregnancy and birth and nonintervention in physiologic processes are among the Hallmarks of Midwifery,^23^ it is probable that the authors’ use of the term advance practice registered nurses (APRNs) was a substitute for nurse‐midwives, although midwives were not mentioned by name or credential anywhere in the publication.

When midwives were explicitly included in the literature, they were often external to the PPC team, and their scope of practice was mispresented. For example, midwives were grouped together with obstetricians as “prenatal care providers,” regarded as solely providing ambulatory care, with a lack of recognition that midwives can provide care throughout the perinatal course and across care settings. The extent of midwives’ education and training was also poorly characterized, with one publication suggesting that midwives involved in PPC might have lower confidence than physicians in providing care due to limited education or experience in dealing with complex diagnoses and conditions.^42^ Although midwives are experts in providing care for low‐risk populations, their education prepares them for collaboration with physicians in team‐based care for individuals with perinatal complications.^45^

Notably, no identified publications mentioned the unique approach to care or scope of practice of midwives. Existing research has shown that individuals classified as “high‐risk” in pregnancy often have difficulty obtaining humanized, low‐intervention care and are more likely to experience anxiety and feelings of vulnerability and loss of autonomy.^46^ Evidence supports that patients with high‐risk conditions or pregnancy‐related complications benefit from the increased support, education, continuity of care, involvement in decision‐making, and person‐ and family‐centered care, which are at the heart of the midwifery model of care.^46^, ^47^ This may be especially true in PPC, with qualitative research concluding that patients and families have unique informational and emotional needs and highly value compassionate caregiving, attentive listening, and validation from care providers.^48^, ^49^ Based on the findings of this scoping review, there is a need for increased awareness and education about the full scope of midwifery practice and the unique benefits of midwifery care in pregnancies with LLFCs.

Many PPC programs have developed within tertiary care settings such as fetal care centers or high‐risk perinatal medicine,^44^ however PPC can (and should) include regional and community hospitals and community‐based providers. Families who desire PPC and opt for a low‐intervention, physiologic approach aligned with the midwifery model of care may prefer to receive care in settings that do not offer the full array of high‐risk specialists and technologies necessary for life‐prolonging interventions. For example, Knowles et al presented a case of a planned community birth with home hospice care.^43^ Although the midwife who provided perinatal care did not directly consult or collaborate with PPC providers, the family was able to work with a PPC team to receive home‐based palliative care and develop a contingency plan for potential complications.^43^ Authors of this case reported that the family feared a hospital would not be able to provide care aligned with their goals and that the midwife's involvement enabled the family's needs to be met by providing family‐centered care in a community birth setting. Fully integrating midwives into PPC teams can expand holistic care options for families and foster continuity of care and bereavement support consistent with the goals of PPC. This is especially beneficial given that existing research has highlighted respect for parent preferences, birth planning, and continual and supportive presence as the most frequently reported important components of PPC.^15^

### Strengths and Limitations

Strengths of this scoping review include its rigorous and transparent process based on a clearly articulated research question and PRISMA Scoping Review (PRISMA‐ScR) guidelines. Multiple research databases were searched to facilitate a comprehensive search of the literature, with additional searches for gray literature and hand‐searching of citations. To reduce the risk of reviewer bias, relevance screening was conducted by multiple interprofessional reviewers. The use of bibliographic reference managing and systematic review software (EndNote and Covidence, respectively) ensured that all identified citations and articles were screened.

The major limitation of this review is that it may not have identified all relevant literature. Although the search strategy was designed to be comprehensive, it is possible that inclusion of additional databases or publications in languages other than English may have expanded the findings. As a scoping review, this study did not assess methodological quality of included studies, and as such, it is possible that conclusions are drawn based on research that lacked methodological rigor. With a small number of identified publications, it is possible that the results do not fully reflect regional differences in midwives’ scope of practice or roles in interprofessional PPC teams.

### Implications

The findings of this review reveal that the role of midwives is either overlooked or insufficiently explored within PPC. Nevertheless, this review of midwives’ roles in PPC introduces relevant implications for clinical practice, midwifery education, health policy, and future research, as described below.

#### Clinical Practice

Midwives are uniquely positioned to provide holistic, family, and person‐centered care in situations of pregnancy with LLFCs and can enhance PPC teams in both the provision of clinical care and care planning and coordination. Optimal PPC includes an individualized, relational decision‐making approach, values clarification, and support for informed choice throughout the perinatal course to ensure alignment with the patient's informational needs, goals, and preferences.^5^, ^29^, ^50^ In the antepartum period, this includes individualized counseling at the time of diagnosis regarding the full spectrum of care options including continuation or termination of pregnancy and any relevant diagnostic testing and prenatal or maternal‐fetal interventions. Aligned with the guidelines developed by Resolve Through Sharing,^50^ PPC should include specialized childbirth education and birth planning that meets families’ unique and complex health and psychosocial needs. Midwives can also facilitate coordination of prenatal care and specialty consultations, which is often complex in cases of LLFCs. Care coordination includes making or accepting referrals, ensuring timely consultations, scheduling testing, and coordinating follow‐up care, as well as communication with families and PPC team members.^15^

In addition to decisions about preferred care common to all birthing people, PPC planning involves individualized decisions around the timing and mode of birth, labor management (including labor induction or augmentation), use of fetal monitoring, and presence of neonatal care providers. Unique postpartum decisions may include location of care, lactation care (including maintenance, suppression, or milk donation), discharge planning and timing, and coordination of follow‐up postpartum care. Decisions must also be made about plans for the newborn including resuscitation and other interventions to promote or sustain neonatal survival, bonding activities, spiritual or religious practices, routine newborn care and infant feeding, testing and monitoring, palliative care efforts to increase comfort and quality of life such as pain and symptom management, and end‐of‐life care planning.^7^ Midwives can facilitate informed decision‐making aligned with families’ preferences and values by engaging in these discussions with the pregnant person, their support people, and other care providers and then working with the PPC team to ensure coordination of care in recognition of those goals. Midwifery care includes providing education, anticipatory guidance, and support to patients and their families in upholding, revisiting, or amending plans as care evolves.

In the immediate postpartum, midwives in PPC facilitate the family's preferences for bonding and holding their newborn and engaging in memory‐making such as photos and handprints, bathing and dressing, and spiritual or religious activities. Midwives should support patients’ coping and grief‐processing and provide referrals and resources to support their emotional and psychological well‐being. They can also facilitate the provision of postmortem care such as autopsy and other testing, organ donation, and funeral arrangements. After the initial postpartum period, ongoing follow‐up and supportive care is beneficial throughout the first year of loss, with special attention to outreach with a phone call and card on the anniversary of the birth/loss. When applicable, resources should be shared with the family to help them talk with their other family members and children.^7^ All of these decisions and discussions require compassionate communication, cultural humility and respect for diversity, and grounding in ethics that honor patient autonomy. Table 4 provides resources for midwives involved in clinical PPC.

**Table 4:** Resources for Midwives Involved in Clinical Perinatal Palliative Care

Resource	Description	Website
AWOHNN's Perinatal Bereavement Program	Resources for perinatal nurses including a training program, checklists, and education videos.	awhonn.org/perinatal‐bereavement‐resources
Now I Lay Me Down to Sleep	Remembrance photography for families experiencing perinatal loss (free professional portraiture).	nowilaymedowntosleep.org
Perinatal Hospice and Palliative Care	Informational clearinghouse including patient and caregiver resources and a list of PPC programs.	perinatalhospice.org
Pregnancy Loss and Infant Death Alliance	Perinatal and neonatal bereavement care organization focused on education, advocacy, and networking for health care providers and parent advocates.	plida.org
Resolve Through Sharing	Education information and resources for bereavement care across the life span, including perinatal death.	resolvethroughsharing.org
Share: Pregnancy Loss Support	Grief support organization for perinatal loss including information and resources for parents and families.	nationalshare.org

Abbreviation: AWOHNN, Association of Women's Health, Obstetric and Neonatal Nurses; PPC, perinatal palliative care.

Midwives are well‐positioned to provide PPC across a variety of care settings, including ambulatory centers, hospitals, and community‐based settings such as home and birth centers. Notably, midwives are increasingly being incorporated into tertiary care settings, such as fetal care centers, which are becoming more prevalent. These specialty centers are hubs for coordinated, multidisciplinary care dedicated to severe and LLFCs throughout the perinatal course.^51^, ^52^ The inclusion of midwives in PPC teams across care settings is essential to support patients’ access to high‐quality, person‐centered perinatal care that aligns with their values and preferred birth setting. Fetal care centers and other PPC programs should recognize the potential enhancement of holistic patient care and PPC effectiveness by integrating midwives into their care teams.

Finally, it is important to acknowledge that all care providers involved in the provision of PPC, including midwives, are at risk for secondary traumatic stress, defined as “the stress resulting from helping or wanting to help a traumatized or suffering person.”^53^ Because of the strong emotions that may accompany a LLFC and perinatal death and the relationship‐centered midwifery model of care, midwives may be at increased risk for secondary traumatic stress in these cases.^42^, ^54^ As Beck, LoGiudice, and Gable reported in a survey of 473 midwives practicing in the United States who attended traumatic births, 29% reported high to severe secondary traumatic stress, with 36% screening positive for posttraumatic stress disorder.^54^ The most common type of traumatic birth that midwives described in this study was fetal demise or neonatal death. Midwives providing PPC should take measures such as debriefing, mentoring, and self‐care activities to foster resilience and prevent secondary traumatic stress, compassion fatigue, and burnout.^55^

#### Midwifery Education

All perinatal health care providers should receive training and continuing education for palliative and bereavement care.^56^, ^57^ Providing PPC and psychosocial support for pregnant persons with LLFCs is included in the ACNM Core Competencies for Basic Midwifery Education that define the requisites for all midwifery graduates from programs accredited by the Accreditation Commission for Midwifery Education.^23^ At present, the Core Competencies include the provision of or referral for options counseling and support for individualized decision‐making for “unplanned or undesired pregnancy.”^23^ However, they do not specify midwifery scope of practice for options counseling or abortion care for planned, wanted pregnancies with fetal anomalies. The ACNM Core Competencies should be revised to recognize the need for options counseling and decision‐making support for individuals facing the emotional and difficult decisions involved in pregnancies with LLFCs. Midwifery education programs should aim to prepare graduates with the knowledge and skills to provide compassionate, person‐centered care for individuals who have a fetus with LLFC and may be considering PPC or termination of pregnancy due to fetal anomaly (TOPFA). This is in alignment with recommendations from the American College of Obstetricians and Gynecologists (ACOG) and American Academy of Pediatrics (AAP) that persons pregnant with a fetus with LLFC should be presented with the full spectrum of care options.^5^, ^51^ Since the Supreme Court's *Dobbs v. Jackson Women's Health Organization* ruling, state restrictions on abortion care limit choices in pregnancies with LLFCs, including access to TOPFA. As a result, it will become increasingly important for midwives and other reproductive health care professionals to be prepared to provide PPC.

Midwifery education programs should ensure that graduates can provide adequate counseling, support informed decision‐making, and facilitate perinatal and bereavement care for persons who continue pregnancies with LLFCs. To enable students to meet required competencies, educators should facilitate clinical or simulated learning opportunities for students to gain skills in perinatal care, counseling, and bereavement care for persons with LLFCs. Educators should support learners in sharing and processing their emotions following these experiences. Simulated clinical encounters with patient actors can prepare students for these challenging conversations and aspects of care.^33^, ^34^ Simulations that include learners from other disciplines such as nursing, obstetrics, pediatrics, social work, and pastoral care are ideal to provide students the opportunity to practice working as a part of an interprofessional care team.^58^ LoGiudice and O'Shea's model may serve as a helpful resource for educators looking to create or to augment curricula to incorporate PPC.^34^ Until care for LLFCs including PPC is comprehensively integrated into all midwifery education programs, midwives may seek continuing education in PPC through specially designed programs such as Association of Women's Health, Obstetric and Neonatal Nurses’ (AWOHNN) Perinatal Bereavement Program or Gundersen Medical Foundation's Resolve Through Sharing perinatal death bereavement training (see Table 4).

#### Health Policy

The intersection of PPC with health policy presents several important considerations for midwifery, underscoring the crucial role of midwives in shaping and championing policies that enhance care and outcomes for individuals facing LLFCs. For example, the absence of standardized quality assessment and certification policies for PPC programs leaves a regulatory void in guiding implementation and assessing outcomes. Since the *Dobbs v. Jackson Women's Health Organization* decision, PPC has increasingly been misrepresented in state and national antiabortion bills as an approach to care for LLFCs that negates the need for TOPFA.^59^ Among the more than 20 states implementing abortion bans since the *Dobbs v. Jackson Women's Health Organization* decision, only 7 include exceptions for LLFCs, and reports are emerging that these exceptions are often ineffective in ensuring access to care.^60^ Other laws misuse PPC to impose barriers to abortion care by mandating that clinicians, including midwives, provide information about PPC as an alternative before TOPFA is legally permitted.^61^

Midwives have a crucial role to play both as clinicians navigating these restrictions on practice and as advocates against policies that harm pregnant people and families navigating LLFCs. Following the *Dobbs v. Jackson Women's Health Organization* decision, the ACNM joined ACOG, AAP, and more than 75 other health care organizations in a statement opposing legislative interference in reproductive health, continuing to support the full range of options for pregnancies with LLFCs, including termination of pregnancy, palliative care, and fetal and/or neonatal intervention.^51^, ^62^ Considering their expertise in person‐centered, comprehensive reproductive care, midwives should play a central role in shaping future policies to ensure the accessibility, effectiveness, and safety of PPC and to honor patients preferences, needs, and values.

#### Research

The lack of original research identified by this scoping review is echoed in other recent literature reviews on the components and outcomes of PPC.^8^, ^63^ More research is needed in this emerging area of clinical practice, and midwives should be encouraged to contribute to the generation of this knowledge and enhance its development with their unique perspective and care philosophy. Specifically, research is needed to optimize strategies to incorporate PPC into midwifery education and identify barriers and facilitators of personal and professional sustainability in providing PPC. Additionally, research on the outcomes of PPC teams that include midwives should be undertaken to explore their distinct contributions and the effects of their involvement in PPC regarding health care quality indicators and perinatal outcomes. Based on the findings of this review, there is a need for increased clarity in future research to describe the roles and contributions of PPC team members.

## CONCLUSION

Although midwives are uniquely positioned to make valuable contributions to high‐quality PPC across care settings in collaboration with specialists from other disciplines, midwives’ role in PPC in the United States at present is not well recognized or explored. A greater emphasis should be placed on incorporating PPC into midwifery curricula and continuing education opportunities to ensure that midwives are adequately prepared to meet the needs of pregnant persons and their families who choose to continue pregnancies with a LLFC, especially as the demand for such care is rapidly increasing. The integration of midwives into PPC programs and services has the potential to enhance accessibility and quality of care. Recognizing the intersection of PPC with reproductive health policy, midwives stand poised to advocate for policies that enhance care and outcomes for individuals facing LLFCs.

## CONFLICT OF INTEREST

Abigail Wilpers has a paid consultant position on the National Faculty of Resolve Through Sharing, Gundersen Medical Foundation, Inc., La Crosse, Wisconsin. The remaining authors declare no conflicts of interest.

## Supporting information


**Appendix S1**. Search Strategy

**Appendix S2**. Preferred Reporting Items for Systematic Reviews and Meta‐Analyses Extension for Scoping Reviews (PRISMA‐ScR) Checklist
