# Supplementary material for: The Role of Midwives in US Perinatal Palliative Care: A Scoping Review
Source: J Midwifery Womens Health. 2024 Jul 9;69(6):875–87. doi: 10.1111/jmwh.13664 (PMC11622358; doi:10.1111/jmwh.13664)
Supplement: Supplementary file 1 — Appendix S1. Search Strategy [file JMWH-69-875-s002.docx]

**Supporting Information Appendix S1**. Search strategy

The search strategy for this study was structured around two distinct blocks: (1) the population (midwives or advanced practice nurses) and (2) the concept (perinatal palliative or hospice care).

An initial search was conducted in PubMed to ensure the relevance of identified studies. The syntax used for this search was then translated and applied to other academic databases: CINAHL (EBSCO), Embase, and Web of Science. ProQuest was searched to identify dissertations and theses. Finally, the search was conducted in Google Scholar, with the first 100 results being included in the review. Results were limited to English language publications. No limitations as to location or date of publication were applied. The citations of all identified reviews and included publications were scanned, and relevant references were then incorporated into the review.

The detailed search strategy for each database was as follows.

1. **PubMed**

((("Hospice Care"[MeSH Terms] OR "Hospices"[MeSH Terms] OR "Hospice and Palliative Care Nursing"[MeSH Terms]) AND "Perinatal Care"[MeSH Terms]) OR ("perinatal palliative care"[All Fields] OR "perinatal hospice"[All Fields])) AND ("midwife*"[All Fields] OR "midwive*"[All Fields] OR "advanced practice nursing"[All Fields] OR ("nursing"[MeSH Terms] OR "nursing"[All Fields] OR "nursings"[All Fields] OR "nursing"[MeSH Subheading] OR "nursing s"[All Fields]) OR ("Midwifery"[MeSH Terms] OR "Nurse Midwives"[MeSH Terms]))

1. **CINAHL (EBSCO)**

S1 (nurse midwife or midwife or midwives or midwifery) OR (advanced practice nurse or advanced practice nursing or nurse practitioners or nursing)

S2 perinatal palliative care or perinatal hospice

S3 (MH "Midwives") OR (MH "Midwife Attitudes") OR (MH "Midwifery Service+")

S4 (MH "Palliative Care") OR (MH "Palliative Care Nursing") OR (MH "Palliative Care Nurses") OR (MH "Palliative Medicine") OR (MH "Hospice Patients") OR (MH "Hospice Nurses") OR (MH "Hospice Nursing") OR (MH "Hospice Care") OR (MH "Hospices")

S5 (MH "Perinatal Period") OR (MH "Perinatal Care")

S6 S4 AND S5

S7 S2 OR S6

S8 S1 OR S3

S9 S7 AND S8

**C. Embase**

“perinatal palliative care” AND midwife

**D. Web of Science**

perinatal palliative care OR perinatal hospice (All Fields) and midwife (All Fields)

**E. ProQuest (Dissertations and Theses Global)**

(perinatal palliative care or perinatal hospice care) AND (nurse midwife or midwife or midwifery or midwives)

**F. Google Scholar**

*Limited to first 100 results*

perinatal (palliative care OR hospice) AND (midwifery OR midwife OR midwives)
